# First Diagnosis and Management of Incontinence in Older People with and without Dementia in Primary Care: A Cohort Study Using The Health Improvement Network Primary Care Database

**DOI:** 10.1371/journal.pmed.1001505

**Published:** 2013-08-27

**Authors:** Robert L. Grant, Vari M. Drennan, Greta Rait, Irene Petersen, Steve Iliffe

**Affiliations:** 1 Faculty of Health, Social Care and Education, Kingston University and St. George's University of London, United Kingdom; 2 Research Department of Primary Care and Population Health, University College London, London, United Kingdom; King's College London, United Kingdom

## Abstract

**Background:**

Dementia is one of the most disabling and burdensome diseases. Incontinence in people with dementia is distressing, adds to carer burden, and influences decisions to relocate people to care homes. Successful and safe management of incontinence in people with dementia presents additional challenges. The aim of this study was to investigate the rates of first diagnosis in primary care of urinary and faecal incontinence among people aged 60–89 with dementia, and the use of medication or indwelling catheters for urinary incontinence.

**Methods and Findings:**

We extracted data on 54,816 people aged 60–89 with dementia and an age-gender stratified sample of 205,795 people without dementia from 2001 to 2010 from The Health Improvement Network (THIN), a United Kingdom primary care database. THIN includes data on patients and primary care consultations but does not identify care home residents. Rate ratios were adjusted for age, sex, and co-morbidity using multilevel Poisson regression.

The rates of first diagnosis per 1,000 person-years at risk (95% confidence interval) for urinary incontinence in the dementia cohort, among men and women, respectively, were 42.3 (40.9–43.8) and 33.5 (32.6–34.5). In the non-dementia cohort, the rates were 19.8 (19.4–20.3) and 18.6 (18.2–18.9). The rates of first diagnosis for faecal incontinence in the dementia cohort were 11.1 (10.4–11.9) and 10.1 (9.6–10.6). In the non-dementia cohort, the rates were 3.1 (2.9–3.3) and 3.6 (3.5–3.8).

The adjusted rate ratio for first diagnosis of urinary incontinence was 3.2 (2.7–3.7) in men and 2.7 (2.3–3.2) in women, and for faecal incontinence was 6.0 (5.1–7.0) in men and 4.5 (3.8–5.2) in women. The adjusted rate ratio for pharmacological treatment of urinary incontinence was 2.2 (1.4–3.7) for both genders, and for indwelling urinary catheters was 1.6 (1.3–1.9) in men and 2.3 (1.9–2.8) in women.

**Conclusions:**

Compared with those without a dementia diagnosis, those with a dementia diagnosis have approximately three times the rate of diagnosis of urinary incontinence, and more than four times the rate of faecal incontinence, in UK primary care. The clinical management of urinary incontinence in people with dementia with medication and particularly the increased use of catheters is concerning and requires further investigation.

*Please see later in the article for the Editors' Summary*

## Abstract 2

Robert Grant and colleagues used the British THIN primary care database to determine rates of first diagnosis of urinary and faecal incontinence among people aged 60–89 with dementia compared with those without dementia, and the use of medication or indwelling catheters for urinary incontinence in those with and without dementia.

*Please see later in the article for the Editors' Summary*

## Editors' Summary

**Background:**

Globally, more than 35 million people have dementia, brain disorders that are characterized by an irreversible decline in cognitive functions such as language and memory. Alzheimer's disease and other forms of dementia mainly affect older people and, because people are living longer than ever, experts estimate that by 2050 more than 115 million people will have dementia. The earliest sign of dementia is usually increasing forgetfulness but, as the disease progresses, people gradually lose their ability to deal with normal daily activities such as dressing, they may become anxious or aggressive, and they may lose control of their bladder (urinary incontinence), bowels (bowel or fecal incontinence), and other physical functions. As a result, people with dementia require increasing amounts of care as the disease progresses. Relatives and other unpaid carers provide much of this care—two-thirds of people with dementia are cared for at home. However, many people with dementia end their days in a care or nursing home.

**Why Was This Study Done?:**

Incontinence in people with dementia is distressing for the person with dementia and for their carers and often influences decisions to move individuals into care homes. However, little is known about the diagnosis and treatment of urinary and/or fecal incontinence among people with dementia living at home. This information is needed to help policymakers commission the services required for this section of society and insurers recognize the needs such patients have, as well as helping to raise clinicians' awareness of the issue. In this cohort study (an investigation that compares outcomes in groups of people with different characteristics), the researchers use data routinely collected from general practices (primary care) in the UK to determine the rate of first diagnosis of urinary and fecal incontinence in elderly patients with and without dementia and to find out whether a diagnosis of dementia affects the rate of use of drugs or of indwelling urinary catheters (tubes inserted into the bladder to drain urine from the body) for the treatment of urinary incontinence.

**What Did the Researchers Do and Find?:**

The researchers extracted data collected between 2001 and 2010 on incontinence for nearly 55,000 people aged 60–89 with a diagnosis of dementia (the dementia cohort) and for more than 200,000 individuals without a diagnosis of dementia (the non-dementia cohort) from The Health Improvement Network (THIN) primary care database, which includes anonymized consultation records from nearly 500 UK general practices. In the dementia cohort, the rates of first diagnosis of urinary incontinence were 42.3 and 33.5 per 1,000 person-years at risk among men and women, respectively. In the non-dementia cohort, the corresponding rates were 19.8 and 18.6. The rates of first diagnosis of fecal incontinence were 11.1 and 10.1 in the dementia cohort, and 3.1 and 3.6 in the non-dementia cohort among men and women, respectively. After adjusting for age, sex and other diseases, the adjusted rate ratio for the first diagnosis of urinary incontinence in people with dementia compared to people without dementia was 3.2 in men and 2.7 in women; for fecal incontinence, it was 6.0 in men and 4.5 in women; the adjusted rate ratio was 2.2 for both men and women for drug treatment of urinary incontinence and 1.6 in men and 2.3 in women for use of indwelling urinary catheters.

**What Do These Findings Mean?:**

These findings indicate that, in primary care in the UK, dementia is associated with a three-fold higher rate of diagnosis of urinary incontinence and a greater than four-fold higher rate of diagnosis of fecal incontinence. Moreover, the authors suggest that some aspects of clinical management of urinary continence vary between people with and without dementia. In particular, the use of indwelling urinary catheters appears to be more common among people with dementia than among people without dementia, increasing the risk of infection. Thus, health care practitioners providing care for people with dementia may be prioritizing ease of management over risk avoidance, a possibility that requires further investigation. Although the accuracy of these findings is limited by certain aspects of the study design (for example, the THIN database does not identify which patients are living in care homes), they nevertheless suggest that policymakers and insurers involved in planning and providing services for people with dementia living at home need to provide high levels of help with incontinence, including the provision of advice and support for carers.

**Additional Information:**

Please access these websites via the online version of this summary at http://dx.doi.org/10.1371/journal.pmed.1001505.

- The UK not-for-profit organization Alzheimers Society provides information for patients and carers about dementia, including information on coping with incontinence and personal stories about living with dementia
- The US not-for-profit organization Alzheimers Association also provides information for patients and carers about dementia and about incontinence, and personal stories about dementia
- The UK National Health Service Choices website provides information (including personal stories) about dementia, urinary incontinence, and bowel incontinence
- MedlinePlus provides links to further resources about dementia, urinary incontinence and fecal incontinence (in English and Spanish)
- The International Continence Society and the International Consultation on Urological Diseases provide independent advice on products to manage incontinence
- More information about the THIN database is available

## Introduction

Dementia is one of the most disabling and burdensome diseases. It is increasing in incidence and prevalence worldwide and has a considerable impact on health and social care systems [1]–[3]. Incontinence is a significant contributor to the burden of those providing unpaid care to people with dementia and often triggers the relocation of people to care homes [4]–[6]. The clinical syndrome of dementia includes progressive deterioration in cognition, the abilities to undertake daily living activities, and physical functioning, including control of voiding and coping with incontinence [7],[8]. In addition, behavioural and psychological problems can manifest in socially inappropriate voiding behaviours [9],[10]. Health professionals, service planners, and policy makers in many countries are seeking ways to support family carers and enable people with dementia to remain in their own homes [11],[12].

There is evidence that older adults delay seeking medical help for incontinence and that the responses of some health professionals to incontinence are sub-optimal [13]–[16]. In addition, older adults are less likely to receive treatment congruent with evidence-based guidelines [16]–[19]. The high prevalence of incontinence in people with dementia who reside in care homes is well documented [8], but international estimates suggest that over two-thirds of all people with dementia live in their own homes [20],[21]. Effective planning and commissioning of services for this population requires data on the incidence of incontinence among people with dementia living at home, yet a recent systematic review identified that there were no published studies that reported the incidence or prevalence of urinary and/or faecal incontinence in this community-dwelling population [22]. One recent study in low- and middle-income countries (China, India, and Latin America) identified an increasing prevalence of incontinence with dementia severity [1].

This study aimed to investigate the first diagnosis and treatment of urinary and faecal incontinence in people with dementia living in the community, by analysing routinely collected data from general practices in the United Kingdom through The Health Improvement Network (THIN) [23]. We sought to answer these questions: (1) What is the rate of first diagnosis of urinary and faecal incontinence in primary care patients aged 60–89 y with diagnosis of dementia compared to those without diagnosis of dementia? (2) In general practice patients aged 60–89 y with a diagnosis of urinary incontinence, does the presence of a diagnosis of dementia, compared to no diagnosis of dementia, affect the rate of first use of (a) pharmacological treatments or (b) indwelling urinary catheters for urinary incontinence?

## Methods

### Data Source

We used data from THIN primary care database. This database comprises routine records of consultations from nearly 500 general practices, the main providers of primary care within the National Health Service (NHS) in the United Kingdom. The NHS provides for universal registration as a patient with a general practice, including residents in care homes (either residential homes or nursing homes) [24]. Over 98% of the UK population are registered with a general practice and THIN has been shown to be representative of national statistics for general practice patients [25]. THIN contains coded records for symptoms, diagnoses, investigations, prescriptions, referrals, demographics, and some neighbourhood characteristics, including socio-economic deprivation as described by quintiles of Townsend score (derived from 2001 Census data for unemployment, overcrowding, car ownership, and home ownership) [26].

### Study Population

We selected a dementia cohort identified by the presence of one or more codes associated with a clear diagnosis of dementia, or two or more prescriptions for drugs unambiguously intended to treat dementia (donepezil, galantamine, memantine, rivastigmine). Lists of these codes were developed by searching data dictionaries using the method described by Davé and Petersen and confirmed by two general practitioners (GR and SI) [27]. We excluded data from the first 4 mo following new registration with a practice, as this has been shown to contain retrospective recording of a past history rather than a true incident recording of a new episode of dementia [28]. Patients in the dementia cohort were followed up from the latest of the following: their 60th birthday, the day they registered with the practice, the day of their first dementia code, the day the practice was deemed to have achieved an acceptable level of data quality [29]–[31], or 1 January 2001. Patients exited the study on the earliest of the following: the day they transferred out of the practice, their date of death, the last date the practice contributed data to THIN, or 31 December 2010. Patients with prevalent incontinence were not at risk of acquiring it, and so were not eligible for the study. Patients with less than 6 mo data in the study, those aged 90 y or over at entry, and those with any codes indicating learning disabilities or Down syndrome were excluded.

For the non-dementia cohort, we identified a random sample of up to four times as many people without a record of dementia, stratified on general practice, 5-y age bands, and sex. In some practices it was not possible to obtain exactly four times as many individuals without dementia in the older age bands.

### Outcomes and Covariates

Incontinence was defined by the presence of one or more diagnostic codes for incontinence, or two or more prescriptions for urinary incontinence (darifenacin, fesoterodine, flavoxate, oxybutynin, propiverine, solifenacin, tolterodine, trospium, and a wide range of devices specifically for absorption or containment). Patients identified as having acquired urinary incontinence were included in analysis for the second research question. The date of the first prescription of a pharmaceutical treatment was taken as the endpoint for research question 2(a). For research question 2(b) on prolonged use of catheters, we sought two or more prescriptions of indwelling urinary catheters within a 6-mo period and took the earliest such date as the endpoint.

A measure of treated co-morbidity was taken for each patient by identifying the number of different drug classes, as defined by the chapters of the British National Formulary [32], from which they had received prescribed drugs in the 6 mo preceding the first incontinence code. For those without incontinence, a 6-mo period was instead selected at random from the participant's time between entering and exiting the study. Drugs relevant to dementia and incontinence were excluded from this count. For analysis, the count was categorised into 0, 1–3, 4–6, 7–9, and >9. We adopted this approach rather than a measure of co-morbidity such as the Charleson index because prescribed drugs not only indicate co-morbidity but also influence treatment decisions through awareness of the risks of polypharmacy [33], and to capture current conditions more reliably than codes for diagnoses may allow. A methodological study comparing different co-morbidity measures in a primary care database of mainly older people concluded that counts of different drugs or drug classes were comparable to more formally validated scales in their ability to predict resource use and serious outcomes [34]. The categorisation allowed for a non-linear relationship between the number of drug classes and the outcomes, in line with similar findings for co-morbidity [35].

### Ethical Review

The NHS South-East Multicentre Research Ethics Committee approved the scheme for THIN to obtain and provide anonymous patient data to researchers, and scientific approval for this study was obtained from THIN Scientific Review Committee in October 2011 [23].

### Statistical Analysis

Rates and rate ratios were calculated for people with and without dementia. Because the data reflect routine records in primary care, these rates quantify the first diagnosis of incontinence, and the first treatment, and not the actual onset of disease. Given the large dataset, approximate 95% confidence intervals were calculated and the Mantel-Haenszel formula was used to pool crude rate ratios. Rates were further analysed by multilevel Poisson regression including the general practice as a random effect [36]. The impact of dementia was adjusted for sex, age, co-morbidity, and quintiles of Townsend score by including them as fixed effects in the regression in that order.

For the second research question about treatments for urinary incontinence, only people with a record of urinary incontinence were considered, and the time period after the first such record was analysed. Multilevel Poisson regression was used to obtain adjusted rate ratios for the first use of pharmaceutical treatment or prolonged use of indwelling catheters. All data extraction and analyses were conducted in Stata software.

## Results

### Characteristics of the Cohorts

We identified 1,246,963 people from THIN aged 60 to 89 y, from 487 practices. Of these, 54,816 entered the dementia cohort. The rate of first diagnosis of dementia rose steadily over that time from 4.2 per 1,000 person-years at risk (PYAR) in 2001 to 4.9 per 1,000 PYAR in 2010, with a spike in 2006, which was reported previously [37]. The sampling for the non-dementia cohort selected 205,795 individuals without incontinence at study entry, which together with the dementia cohort made a total of 1,228,777 PYAR for analysis in the first research question; five people were excluded because the endpoint occurred on the day they entered the study. Urinary incontinence codes were acquired by 8,987 people (16%) in the dementia cohort and 23,083 in the non-dementia cohort (11%), and these people's records were analysed for the second research question on treatments, making a total of 92,173 PYAR. The analysis of pharmacological treatments excluded 7,897 people, and the analysis of catheters excluded 812 people, because the endpoint occurred on the day they entered the study. Faecal incontinence codes were acquired by 2,909 (5%) and 4,784 (2%), respectively, and double incontinence by 420 (0.7%) and 240 (0.1%), respectively. Further details of demographics are shown in Table 1.

**Table 1:** Characteristics of the cohorts.

Characteristics	Category/Statistic	Dementia Cohort (*n* = 54,816)		Non-dementia Cohort (*n* = 205,795)	
		Men (33%, *n* = 18,187)	Women (67%, *n* = 36,629)	Men (34%, *n* = 70,925)	Women (66%, *n* = 134,870)
Age at entry to study	Median (IQR)	78 (72–83)	81 (75–85)	77 (72–82)	80 (75–84)
Years in the study	Median (IQR)	4.5 (2.0–8.0)	4.5 (2.1–7.9)	5.3 (2.5–8.8)	5.6 (2.6–9.0)
Months from first dementia code to first incontinence code	Median (IQR)	9 (0–25)	11 (0–30)	N/A	N/A
Townsend deprivation	1 (Least deprived)	4,173 (23%)	8,323 (23%)	16,648 (23%)	28,347 (21%)
	2	4,169 (23%)	8,212 (22%)	16,031 (23%)	29,587 (22%)
	3	3,822 (21%)	7,802 (21%)	14,253 (20%)	28,176 (21%)
	4	3,328 (18%)	6,859 (19%)	12,799 (18%)	26,227 (19%)
	5 (Most deprived)	2,067 (11%)	4,214 (12%)	8,688 (12%)	17,707 (13%)
	Missing	628 (3%)	1,219 (3%)	2,506 (4%)	4,826 (4%)
Co-morbidity index	No drugs	1,914 (11%)	3,177 (9%)	9,461 (13%)	15,631 (12%)
	1–3 classes	3,859 (21%)	7,885 (22%)	16,959 (24%)	29,503 (22%)
	4–6 classes	5,650 (31%)	11,676 (32%)	20,762 (29%)	39,536 (29%)
	7–9 classes	4,165 (23%)	8,637 (24%)	14,035 (20%)	29,368 (22%)
	10 or more classes	2,599 (14%)	5,254 (14%)	9,708 (14%)	20,832 (15%)

IQR, inter-quartile range; N/A, not applicable.

### Diagnosis of Incontinence

**Figure 1:**
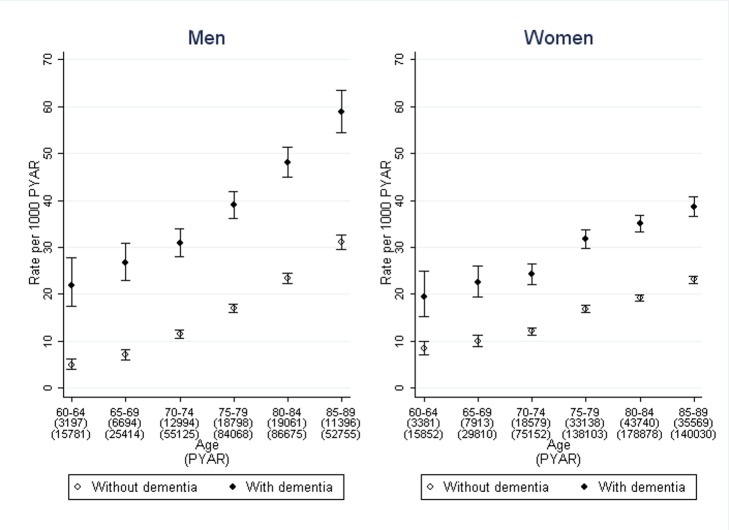
Rates of first diagnosis of incontinence in men and women with dementia compared to those without. Bars indicate 95% confidence intervals.

The crude rate of first diagnosis per 1,000 PYAR of any type of incontinence across both cohorts rose steadily from 19.4 in 2001 to 27.0 in 2010. The rates per 1,000 PYAR (95% CI) for urinary incontinence in the dementia cohort were 42.3 (40.9–43.8) in men and 33.5 (32.6–34.5) in women. In the non-dementia cohort, the rates were 19.8 (19.4–20.3) in men and 18.6 (18.2–18.9) in women (Figure 1). The rates per 1,000 PYAR for faecal incontinence in the dementia cohort were 11.1 (10.4–11.9) in men and 10.1 (9.6–10.6) in women. In the non-dementia cohort, the rates were 3.1 (2.9–3.3) in men and 3.6 (3.5–3.8) in women. Among those with records of both dementia and incontinence, the median time between these records was 9 mo (inter-quartile range 0–25 mo) in men and 11 mo (inter-quartile range 0–30 mo) in women.

After adjustment for age, sex, and co-morbidity, the rate ratio of urinary incontinence, comparing those with dementia to those without, was 3.2 (95% CI 2.7–3.7) in men and 2.7 (2.3–3.2) in women (Table 2). For faecal incontinence, the adjusted rate ratio was 6.0 (95% CI 5.1–7.0) in men and 4.5 (3.8–5.2) in women. Further adjustment by social deprivation did not significantly improve the regression model of association between dementia and either form of incontinence.

**Table 2:** Rate ratios, comparing the dementia and non-dementia cohorts.

Outcome	Adjusted for Age and Sex (95% CI)	Adjusted for Age, Sex and Co-morbidity (95% CI)
First diagnosis of incontinence (any type, including unknown)		
(men)	2.15 (2.06–2.24)	3.19 (2.74–3.70)
(women)	1.80 (1.75–1.86)	2.69 (2.32–3.11)
First diagnosis of urinary incontinence		
(men)	2.13 (2.04–2.22)	3.17 (2.71–3.71)
(women)	1.81 (1.75–1.87)	2.70 (2.31–3.15)
First diagnosis of faecal incontinence		
(men)	3.79 (3.46–4.15)	5.95 (5.06–7.00)
(women)	2.82 (2.64–3.00)	4.48 (3.84–5.21)
First use of pharmacological treatment		
(both sexes)	2.16 (1.31–3.55)	2.23 (1.36–3.67)
First use of prolonged indwelling urinary catheterisation		
(men)	1.36 (1.24–1.49)	1.56 (1.31–1.86)
(women)	1.98 (1.77–2.21)	2.32 (1.94–2.78)

### Association of Dementia Diagnosis with Time to Drug Treatment for Urinary Incontinence

**Figure 2:**
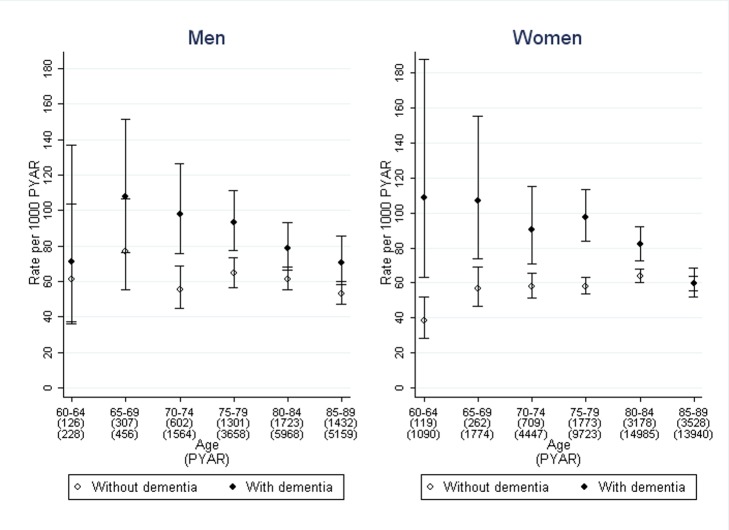
Rates of first use of pharmacological treatment for urinary incontinence in men and women with dementia compared to those without. Bars indicate 95% confidence intervals.

Drug treatment for urinary incontinence was prescribed in 1,309/8,987 (15%) of the people with dementia and 4,223/23,083 (18%) of those without (Figure 2). The median time from recording urinary incontinence to drug treatment in months was: 16 (inter-quartile range 7–31) in men with dementia, 21 (7–47) in men without dementia, 19 (7–38) in women with dementia, 30 (11–66) in women without dementia.

The stratified rates of first pharmacological treatment are shown in Figure 2. After adjustment for age, sex, and co-morbidity, the rate ratio of first pharmacological treatment for urinary incontinence was 2.2 (95% CI 1.4–3.7), comparing those with dementia to those without, with no significant difference between men and women (Table 2). This difference between dementia and non-dementia cohorts was greater in younger age groups: the rate ratio was 2.2 (95% CI 1.4–3.7) for ages 60–64 compared to 1.1 (1.0–1.3) for ages 85–89. Further adjustment by social deprivation did not have an impact on the rate ratios.

### Association of Dementia Diagnosis with Use of Indwelling Urinary Catheters

The rate of first use of prolonged catheterisation was analysed in 12,224 people from the dementia cohort and 34,842 people from the non-dementia cohort, the other 812 having had prolonged indwelling urinary catheters prior to entering the cohorts, making a total of 163,735 PYAR. The median time from incontinence to prolonged use of indwelling urinary catheters in months was: 18 (inter-quartile range 8–37) in men with dementia, 25 (8–58) in men without dementia, 26 (11–48) in women with dementia, and 41 (17–79) in women without dementia.

**Figure 3:**
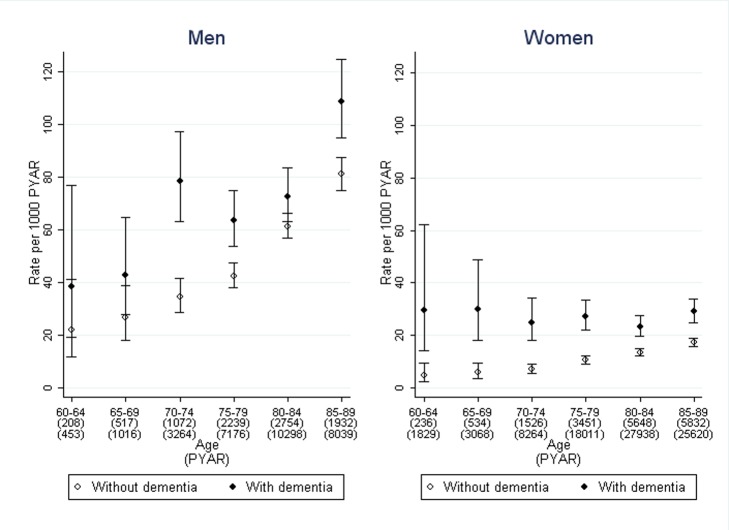
Rate of first use of prolonged indwelling urinary catheterisation in men and women with dementia compared to those without. Bars indicate 95% confidence intervals.

The stratified rates are shown in Figure 3. After adjustment for age, sex, and co-morbidity, the adjusted rate ratio of prolonged catheterisation was 1.6 (95% CI 1.3–1.9) in men and 2.3 (1.9–2.8) in women, comparing those with dementia to those without (Table 2). The rate rises with age and co-morbidity. The regression analysis showed that age, sex, and co-morbidity were significant covariates, along with interactions between co-morbidity and dementia. Only one of the quintiles of Townsend deprivation score was significantly different to baseline but without a clear trend so it was not included as a confounder.

## Discussion

To our knowledge, this the first study to report the rate of first diagnosis of incontinence in primary care in a large community cohort of people with dementia. The presence of a dementia diagnosis was associated with an increased rate of first diagnosis of incontinence in community dwelling people aged 60–89, around double the rate for urinary incontinence, and triple the rate for faecal incontinence compared to people without dementia. This rate ratio, which quantifies the impact of the dementia diagnosis on incontinence rates, was higher in men and increased with age, in line with previous research findings [38]. Adjustment for sex and age led to slightly higher rate ratios and when co-morbidity was also adjusted for, the rate ratios increased notably. The rate ratios were greatest for patients who were younger and who had fewer co-morbidities. This finding reflects the increased risk of incontinence with age and co-morbidity, even in the absence of dementia [7].

The relatively short time from the first recording of dementia to that of incontinence may reflect the late recording of a diagnosis of dementia, which has been previously reported [38], when the risk of incontinence is higher. It may also reflect the known reluctance to seek medical help for continence problems [14],[15]. Family carers of people with dementia have described in some instances only seeking help from health professionals about incontinence problems at the point of crisis [13].

People with dementia and urinary incontinence but no other co-morbidities received drug treatments at more than double the rate for people of the same sex, age, and co-morbidity without dementia. This finding suggests that people with dementia were being offered drug treatments for their urinary incontinence earlier and/or in greater numbers than their counterparts without dementia. This finding requires further investigation to understand the clinical reasoning. This ratio was notably greater for relatively younger people with fewer co-morbidities, which may reflect concerns about drug interactions or polypharmacy that inhibit the use of this form of treatment in the older age groups.

The rate at which use of indwelling urinary catheters was initiated was also higher in people with dementia than people without: 56% greater in men and more than double the rate in women of the same age and co-morbidity. This finding suggests that an older person with dementia was much more likely to receive a urinary catheter, and to receive it sooner, than their counterpart without dementia. Like drug treatment, this difference was attenuated with increasing co-morbidities although it was not affected by age. Unlike drug treatments, there is a less powerful clinical rationale for catheterising people with dementia sooner. Indwelling catheters are associated with increased risk of infection in older people living in their own homes and in nursing homes [39]–[41]. Long term catheter use is known to be associated with discomfort, meatal tissue damage, bladder spasm, and formation of bladder calculi [42]. In addition, the International Continence Society recommends that cognitive problems are considered as a factor to discourage the use of indwelling urinary catheters “through the danger of interference to the catheter” [42]. Given the well-documented risks associated with indwelling catheters, this finding may indicate that ease of management is prioritised over risk avoidance; this requires further investigation.

The strength of this study is the size of the cohorts. We were able to include 54,816 people with a record of dementia, while most studies of additional problems and symptoms in people with dementia have sample sizes in the hundreds [43]–[45]. There are a number of limitations to this study. Care home residents are not routinely identified in THIN, which precluded comparison with individuals resident in their own home [46]. We were unable to investigate surgical treatments for urinary incontinence as the dataset does not contain all information on hospital-based care. In the United Kingdom, NHS-funded absorbent pads are largely supplied through community nursing services rather than general practices, so we have no information on these conservative management strategies. The use of prescriptions to identify patients with urinary incontinence or dementia is not validated and so some uncertainty is attached to it. Because prescriptions for urinary incontinence were used both to identify patients with incontinence and as an outcome, the prescription rates may have been slightly inflated. However, in this study there were no patients identified by dementia drugs who did not also have diagnostic codes, so these rates would not have been influenced by this method. Without data from secondary care and about refusal of referrals, we were unable to determine how many men received indwelling urinary catheters because of urinary obstruction where surgery was not appropriate.

Dementia and incontinence are both conditions in which patients delay seeking help from health professionals so the age at first record in THIN is likely to be greater than the true age at onset of the condition [14],[15]. Finally, the median times between first diagnosis of dementia and first diagnosis of incontinence (or incontinence and treatment) are based on those people who were registered with a general practitioner (GP) and in the cohort during the whole of the time period of interest. This potentially underestimates the population medians by excluding those who acquired dementia or incontinence prior to changing GPs and hence joining THIN, or before 2001.

### Conclusion

The rate of first diagnosis of any type of incontinence is considerably higher among adults aged 60–89 y with dementia than among people of the same age and sex distribution without dementia. Incontinence is a common problem for community-dwelling people with dementia. Providers and planners of services for dementia should anticipate high levels of need, including advice and support for carers managing incontinence. Some aspects of clinical management of urinary incontinence are different for patients with dementia compared with those without. Further study is required to understand the clinical reasoning of health care practitioners providing care for this population, particularly in the use of indwelling catheters, given the known risks.

## Supporting Information

Text S1 **Read codes identifying dementia.**
(DOC)

## Abbreviations

NHS: National Health Service
PYAR: person-years at risk
THIN: The Health Improvement Network (UK)
