# Supplementary material for: First Diagnosis and Management of Incontinence in Older People with and without Dementia in Primary Care: A Cohort Study Using The Health Improvement Network Primary Care Database
Source: PLoS Med. 2013 Aug 27;10(8):e1001505. doi: 10.1371/journal.pmed.1001505 (PMC3754889; doi:10.1371/journal.pmed.1001505)
Supplement: Text S1 — Read codes identifying dementia. (DOC) [file pmed.1001505.s001.doc]

**Supplementary information for "First diagnosis and management of incontinence in older people with and without dementia in primary care: a cohort study using The Health Improvement Network primary care database"**

Information on Read codes can be found at <http://systems.hscic.gov.uk/data/uktc/readcodes>

**Read codes identifying dementia**

description readcode

H/O: dementia 1461.00

Senile dementia E00..11

Senile/presenile dementia E00..12

Uncomplicated senile dementia E000.00

Presenile dementia E001.00

Uncomplicated presenile dementia E001000

Presenile dementia with delirium E001100

Presenile dementia with paranoia E001200

Presenile dementia with depression E001300

Presenile dementia NOS E001z00

Senile dementia with depressive or paranoid features E002.00

Senile dementia with paranoia E002000

Senile dementia with depression E002100

Senile dementia with depressive or paranoid features NOS E002z00

Senile dementia with delirium E003.00

Arteriosclerotic dementia E004.00

Multi infarct dementia E004.11

Uncomplicated arteriosclerotic dementia E004000

Arteriosclerotic dementia with delirium E004100

Arteriosclerotic dementia with paranoia E004200

Arteriosclerotic dementia with depression E004300

Arteriosclerotic dementia NOS E004z00

Dementia in conditions EC E041.00

[X]Dementia in Alzheimer's disease Eu00.00

[X]Dementia in Alzheimer's disease with early onset Eu00000

[X]Presenile dementia,Alzheimer's type Eu00011

[X]Primary degen dementia, Alzheimer's type, presenile onset Eu00012

[X]Alzheimer's disease type 2 Eu00013

[X]Dementia in Alzheimer's disease with late onset Eu00100

[X]Alzheimer's disease type 1 Eu00111

[X]Senile dementia,Alzheimer's type Eu00112

[X]Primary degen dementia of Alzheimer's type, senile onset Eu00113

[X]Dementia in Alzheimer's dis, atypical or mixed type Eu00200

[X]Dementia in Alzheimer's disease, unspecified Eu00z00

[X]Alzheimer's dementia unspec Eu00z11

[X]Vascular dementia Eu01.00

[X]Arteriosclerotic dementia Eu01.11

[X]Vascular dementia of acute onset Eu01000

[X]Multi-infarct dementia Eu01100

[X]Predominantly cortical dementia Eu01111

[X]Subcortical vascular dementia Eu01200

[X]Mixed cortical and subcortical vascular dementia Eu01300

[X]Other vascular dementia Eu01y00

[X]Vascular dementia, unspecified Eu01z00

[X] Unspecified dementia Eu02z00

[X] Presenile dementia NOS Eu02z11

[X] Primary degenerative dementia NOS Eu02z13

[X] Senile dementia NOS Eu02z14

[X] Senile dementia, depressed or paranoid type Eu02z16

[X]Delirium superimposed on dementia Eu04100

Alzheimer's disease F110.00

Alzheimer's disease with early onset F110000

Alzheimer's disease with late onset F110100

[X]Other Alzheimer's disease Fyu3000

**Read codes identifying incontinence**

H/O: stress incontinence 1593.00

Double incontinence 16F..00

Incontinent of faeces 19E3.00

Incontinent of faeces symptom 19E3.11

Incontinence of urine 1A23.00

Stress incontinence 1A24.00

Stress incontinence - symptom 1A24.11

Urgency 1A25.00

Urgency of micturition 1A25.11

Urge incontinence of urine 1A26.00

Urge to pass urine again shortly after finishing voiding 1A27.00

Pis en deux 1A27.11

Terminal dribbling of urine 1A36.00

Dribbling of urine 1A37.00

Smells of urine 222K.00

Bowels - continence 393..11

Bowels-incontinence assessment 393..12

Bowels: incontinent 3930.00

Bowels: occasional accident 3931.00

Bladder-incontinence assessmnt 394..11

Bladder- continence assessment 394..12

Bladder: incontinent 3940.00

Bladder: occasional accident 3941.00

Continence assessment 39H..00

Continence reassessment 39H0.00

Health education - continence 679H.00

Promotion of continence 679H.11

Colposuspension of bladder neck 7B31200

Burch colposuspension 7B31211

Insertion retropubic device stress urinary incontinence NEC 7B33800

Insertion retropubic dev fem stress urinary incontinence NEC 7B33C00

Insertion of bulbar urethral prosthesis 7B42100

Insertion of Kaufman prosthesis for male incontinence 7B42111

Insertion of prosthesis for compression bulb of male urethra 7B42112

Insertion of Rosen prosthesis for male incontinence 7B42113

Colporrhaphy and amputation of cervix uteri 7D17.11

Incontinence care 8C14.00

Continence care 8C14.11

Urinary bladder control 8D7..00

Bladder control 8D7..11

Incontinence control 8D7..12

Incontinence control 8D71.00

Indwelling urethral catheter 8D74.00

Catheter in situ 8D74.11

Penile sheath provision 8D75.00

Urinary bladder control NOS 8D7Z.00

Bladder training 8E97.00

Bladder drill 8E97000

Referral to continence nurse 8H7w.00

Refer to Urodynamic studies 8HR6.00

Referral to incontinence clinic 8HTX.00

Seen by continence nurse 9Nl8.00

Stress incontinence K198.00

Stress incontinence - female K586.00

[X]Other specified urinary incontinence Kyu5A00

[D]Incontinence of faeces R076.00

[D]Sphincter ani incontinence R076100

[D]Incontinence of faeces NOS R076z00

[D]Incontinence of urine R083.00

[D]Urethral sphincter incontinence R083100

[D] Urge incontinence R083200

[D]Incontinence of urine NOS R083z00

[D] Urgency of micturition R086200

Assisting with toileting Z121400

Procedures to aid continence Z1J..00

Taking the patient to toilet Z1J1.00

Attaching penile sheath Z1J2.00

Putting incontinence pad on Z1J3.00

Changing incontinence pad Z1J4.00

Toileting regimes Z1S..00

Regular toileting Z1S1.00

Planned voiding Z1S2.00

Planned voiding two hourly Z1S2100

Ability to perform toileting activities Z89A.00

Does not perform toileting activities Z89A100

Difficulty performing toileting activities Z89A200

Unable to use urine bottle Z89AE00

Urinary catheter appliance procedures Z915.00

Urinary catheter care Z915.11

Under care of continence nurse ZL22400

Referral to continence nurse ZL62400

Seen by continence nurse ZLA2400

Discharge by continence nurse ZLD7400

Bowels incontinence assessment ZQ3C.00

OPCS continence disability scale ZRas400
